# The Influence of Speech-Language-Hearing Therapy Duration on the Degree of Improvement in Poststroke Language Impairment

**DOI:** 10.1155/2017/7459483

**Published:** 2017-01-11

**Authors:** Hitoshi Hayashi, Eisaku Okada, Yosuke Shibata, Mieko Nakamura, Toshiyuki Ojima

**Affiliations:** Department of Community Health and Preventive Medicine, Hamamatsu University School of Medicine, Hamamatsu, Japan

## Abstract

*Background*. The relevance of speech-language-hearing therapy (ST) duration to language impairment remains unclear.* Objective.* To determine the effect of ST duration on improvement in language impairment as a stroke sequela and to compare the findings with those for occupational therapy (OT) and physical therapy (PT).* Methods*. Data regarding patients with stroke sequelae who were registered in the Japanese Association of Rehabilitation Medicine database were analyzed. Propensity scores for ST, OT, and PT duration were calculated using logistic regression, followed by inverse probability weighting in generalized estimating equations to examine the odds ratio for improvement in the Functional Independence Measures scores for comprehension, expression, and memory. Analyses stratified by age and dementia severity were also conducted.* Results*. Compared with short-duration ST, long-duration ST was significantly associated with improved scores for comprehension and expression in the overall study population and in some groups, with higher benefit especially for younger participants (<64 years) and those with more severe dementia. A significant but less pronounced effect was also observed for OT and PT.* Conclusion*. Long-duration ST is more effective than long-duration OT or PT for improving language impairment occurring as stroke sequela. However, these effects are limited by age and severity of dementia.

## 1. Introduction

A large number of studies on rehabilitation training for stroke sequelae have been performed worldwide [1–3]. However, aside from a few studies primarily from Europe and North America, the relevance of the length of speech-language-hearing therapy (ST) with respect to mitigation of poststroke language sequelae has rarely been investigated [4–6], and many issues remain to be clarified. A recent Cochrane Review by Brady et al. [4] evaluated the impact of intensity (e.g., hours per week), dose (i.e., total number of hours), and duration (i.e., total number of weeks/months of intervention) of language therapy. The comparison of tens of randomized clinical trials revealed that a higher treatment dose provided significant improvements only for functional communication, with no consistent effect on other domains (expression, comprehension, and severity). Similarly, few studies showed that longer-duration ST yielded slightly better outcomes in functional communication and receptive language, with no significant effect for expressive language. Furthermore, Brady et al. concluded that the quality of evidence for these effects is rather low. Moreover, it is important to consider that the influence of different parameters (intensity, dose, and duration) cannot be separated easily. This was confirmed, for example, by Pulvermüller et al. [7], who showed that shorter but high-intensity therapy was more effective than the traditional approach involving long-duration but low-intensity therapy amounting to the same total number of hours of treatment (dose). Similarly, a review by Cherney [8] highlighted the fact that the diverse nature of parameters that define treatment intensity makes it difficult to establish decisive recommendations on treatment dose and that the simple notion of “more is better” has not been supported by the evidence.

In some cases, occupational therapy (OT) for impaired upper limb function and physical therapy (PT) for impaired walking function and standing balance are prescribed simultaneously with ST for language impairment. Similar to ST, OT and PT typically involve one-on-one training with the patient. It is believed that the patient experiences a similar benefit from receiving training instructions verbally and participating in natural conversation during OT and PT to that from receiving instructions during ST, and these interactions likely influence the degree of improvement in language impairment. Thus, to determine the need for prescribing ST, it is important to know whether the effects of OT and PT are similar to those of ST in terms of mitigating language impairment after stroke. However, few studies have separately and simultaneously investigated the influences of ST, OT, and PT duration on language impairment [9–12].

Human communication involves several processing steps from comprehension to expression of language. Sound originating from the speaker is first assessed in Wernicke's area (receptive language), and the information is then transferred by the arcuate fasciculus to Broca's area, where expressive language is generated. The information is subsequently sent from Broca's area to the motor cortex to be expressed as speech [13–16]. It is further important to consider that the entire process depends on memory. Therefore, human communication is a complex phenomenon vulnerable to dysfunction. The aging of society, which is a global trend most prevalent in developed countries, poses significant challenges for the fields of speech and language therapy, as elderly individuals have a higher incidence of stroke and thus a higher risk of experiencing poststroke language impairment. It is unclear whether the influence of ST on language impairment is comparable between young and elderly individuals. For example, the study by Laska et al. [5] did not find that age had a significant effect on the outcome when included in a multivariate analysis. Additionally, there has been an increase in the number of dementia patients [17]. In rehabilitation training, dementia reduces the patient's ability to receive training instructions from the therapist. It is important to determine the most appropriate duration of ST for elderly patients and for patients with dementia, in order to ensure appropriate distribution of therapeutic resources, estimate the expenses of patients who have to pay for medical treatment, and evaluate the need for rehabilitation therapy.

The present study evaluated the influence of ST duration on the degree of improvement in language impairment that occurs as a sequela of stroke. In addition, we investigated the influence of OT and PT duration on the degree of improvement in language impairment and compared the findings to those noted for ST. Moreover, we performed further analyses with patients stratified by age and severity of dementia.

## 2. Materials and Methods

### 2.1. Subjects

The Japan Association of Rehabilitation Database includes quality data collected, curated, and maintained as a foundation for advancing science. The database includes data of stroke patients and patients with femoral neck fracture or spinal cord injury, who were treated at a hospital and then transferred to a rehabilitation center for training. We used data registered from 33 hospitals between 2005 and 2013. We extracted the data regarding stroke patients and excluded deficient or incorrect data. The following data were extracted for each patient: demographic characteristics including age and sex; type of rehabilitation training (ST, OT, and PT); rehabilitation training duration; and Functional Independence Measures (FIM) scores [18–20] for comprehension, expression, and memory at hospitalization and discharge. A total of 3,551 stroke patients were finally included in the present study. Information regarding the patients was collected from the medical records maintained at the participating hospitals. The study sample had a high number of patients of relatively advanced age, which is indicative of the high proportion of elderly individuals in Japan. The use of the database received ethical approval from the Japanese Association of Rehabilitation Medicine.

### 2.2. Variables and Assessments

Of the five items included in the FIM cognitive assessment instrument, we selected comprehension, expression, and memory as objective variables describing language impairment. To assess the effect of the therapy on language impairment, the FIM cognitive items related to language function (comprehension, expression, and memory) were assessed in terms of the change in score between admission and discharge, thus indicating whether the patient's condition had improved, worsened, or remained the same after rehabilitation training. These changes were expressed as binary variables, distinguishing between categories of patients defined based on the effect of the therapy.

Binary variables were also used to represent the duration of ST, OT, and PT. In the current sample, the 25%, 50%, and 75% percentiles of treatment dose were 1.3, 22.3, and 53.7 hours for ST; 29.0, 56.0, and 90.0 hours for OT; and 28.0, 58.0, and 99.0 hours for PT, respectively. The 25%, 50%, and 75% percentiles of total treatment duration (all three therapies combined) per patient were 72.0, 141.7, and 239.0 hours, respectively. Based on integer value immediately following the median value, the durations of ST, OT, and PT were converted into binary variables indicative of treatment duration to serve as explanatory variables: <23 versus ≥23 hours, <56 versus ≥56 hours, and <58 versus ≥58 hours, respectively.

Additionally, we used the following factors as covariates: age; sex; hospital; year of hospitalization; level of deficiency in activities of daily living, according to the standardized assessment manual for the elderly with dementia [21, 22] at hospitalization (henceforth referred to as “dementia level”); the Japan Coma Scale [23] score at hospitalization; FIM scores for locomotion (walking/wheelchair use) at hospitalization; FIM scores for comprehension, expression, and memory at hospitalization; duration of hospital (days); and side of the body with more severe paralysis. The various FIM items were treated separately; for example, regarding the change in FIM scores for comprehension, the FIM score for comprehension at hospitalization was used as a covariate. In a limited number of patients, we also administered the Mini-Mental State Examination (MMSE) and confirmed the level of dementia; however, the MMSE scores were not included as covariates.

Patients were divided according to age into the following four groups: ≤54 years, 55–64 years, 65–74 years, and ≥75 years. Patients were divided according to hospitalization year into 2009 or earlier and 2010 or later. According to the dementia level, we divided the patients into the following four groups: normal, mild-to-moderate, severe, and unknown. Specifically, grades 1–2b (mild-to-moderate dementia) describe patients who show some form of dementia but are still largely independent in daily life, both at home and in a social setting, as well as patients who have some disabling symptoms, behaviors, or communication difficulty, even at home, but are still able to act independently as long as appropriate assistance is provided. Grades 3a–M (severe dementia) describe patients who sometimes exhibit disabling symptoms, behaviors, and communication difficulties and require assistance, as well as patients who exhibit significant psychiatric symptoms, behavioral problems, or serious physical disabilities and require specialized medical care.

Patients were divided according to the Japan Coma Scale score as follows: normal, single-digit, double-digit, triple-digit, and unknown scores. Single-digit scores denote patients who are awake even without stimulation. Double-digit scores denote patients who can awaken when stimulated. Triple-digit scores denote patients who do not awake when stimulated (comatose).

Patients were divided according to the FIM locomotion (walk/wheelchair) score at hospitalization as follows: score of 1-2, 3–5, and 6-7 and unknown. Patients having a score of 1-2 cannot walk for more than 15 m, those having a score of 3–5 can walk with assistance, and those having a score of 6-7 can walk unassisted. The patients were also stratified according to FIM scores for comprehension, expression, and memory at hospitalization: score of 1-2, 3–5, and 6-7 and unknown.

According to the length of hospitalization (number of days spent in the hospital) expressed using binary variables, the patients were divided into two groups: ≤91 days and ≥92 days. Additionally, patients were divided according to the side with more severe paralysis as follows: 1, right side; 2, left side; and 1.5, unknown.

### 2.3. Statistical Analysis

We developed propensity scores [24] using a logit model and inverse probability weighting method [25] to compare between the effects of short-duration and long-duration ST, OT, and PT. The concept of propensity scores was originally used to adjust for causal assignment bias in nonrandomized treatment and observational studies. The propensity used score in the present study is the probability of being assigned to one of two groups.

In order to develop the propensity scores, ST, OT, and PT data were analyzed using logistic regression analysis, and the response to long- versus short-duration training was included as an objective variable, while various covariates were included as explanatory variables. Subsequently, we used these propensity scores as explanatory variables to examine changes in comprehension, expression, and memory scores as objective variables and determined the odds ratios (ORs) with 95% confidence intervals (CIs) by inverse probability weighting using a generalized estimation equation. Robust estimation was performed in this assessment.

To calculate the weighting variable, the inverse sampling probability of each individual in the sample was divided by the mean inverse sampling probability of all individuals. This yielded a weighting variable that was scaled such that the mean weight of all individuals would be 1, while the weighted sample size would be equal to the actual, unweighted sample size.

We performed similar analyses after stratification according to age and dementia level in order to investigate the significance of the duration of rehabilitation in elderly patients and in patients with dementia complications. To confirm the validity of the assessment of dementia level, we analyzed its correlation with the MMSE [26] scores using Spearman's correlation coefficient, after excluding patients with unknown scores. Moreover, we calculated the mean values and 95% CIs of the MMSE for patients that were categorized as having normal, mild-to-moderate, severe, and unknown levels of dementia.

All statistical analyses were performed using SPSS version 24.0 (IBM Corp., Armonk, NY), and a *p* value of <0.05 was considered statistically significant. The results of the analyses are expressed in terms of ORs, 95% CIs, and *p* values.

## 3. Results

The basic characteristics of the patients are presented in Table 1. In terms of age, there were a large number of elderly patients, with 66% of patients aged over 65 years. There were slightly more men than women in our sample population. Regarding the level of deficiency in activities of daily living, the patients were distributed fairly evenly among the four categories defined based on dementia level. In terms of stroke classifications, about half of the patients had suffered cerebral infarction, about one-quarter had intracranial hemorrhage, and only a small number had a subarachnoid hemorrhage or some other condition. In terms of the duration of hospitalization, about half of the patients spent less than 91 days in the hospital. About one-third of patients had more severe paralysis on the right side, and another third had more severe paralysis on the left side; in the remaining patients, the side of more severe paralysis was unknown.

An overview of the influence of different ST, OT, and PT duration on the degree of improvement in FIM comprehension score is presented in Table 2 in the form of ORs and 95% CIs and stratified by age and dementia level. In the overall analysis, the degree of improvement in FIM comprehension score was greater with long-duration ST than with short-duration ST. In the age-stratified analysis, the degree of improvement did not differ significantly with the duration of ST among the older patients (age groups ≥75 and 65–74 years); however, ST duration affected FIM comprehension scores significantly among the younger patients (age groups 55–64 and ≤54 years). In the analysis stratified by dementia level, the degree of improvement was greater with long-duration ST than with short-duration ST only in the patients with the most severe dementia. On the other hand, the influence of OT duration on the degree of improvement in FIM comprehension scores was significant in the overall analysis and in the younger group (55–64 years) only, while the influence of PT duration was not significant in any of the comparisons.

An overview of the influence of different ST, OT, and PT duration on the degree of improvement in FIM expression scores is presented in Table 3. In the overall analysis, the degree of improvement in FIM expression was marginally greater with long-duration ST than with short-duration ST. In the age-stratified analysis, the degree of improvement was similar for short- and long-duration ST in older patients (age groups ≥75 and 65–74 years). However, the two durations of ST yielded significantly different outcomes among the younger patients (age groups 55–64 and ≤54 years). In the analysis stratified by dementia level, the degree of improvement was similar across the different levels of dementia. The influence of long-duration OT on the degree of improvement in FIM expression scores was significant only among the youngest patients (≤54 years). On the other hand, the effect of long-duration PT was significant among the oldest patients (≥75 years) and among patients with mild-to-moderate dementia.

An overview of the influence of different ST, OT, and PT duration on the degree of improvement in FIM memory scores is presented in Table 4. In the overall analysis, the degree of improvement in FIM memory was similar for the short- and long-duration ST. In the age-stratified analysis, the degree of improvement was marginally greater with long-duration ST than with short-duration ST among patients aged ≤54 years, while no differences were observed in the other age groups. In the analysis stratified by dementia level, the degree of improvement was significantly greater for long-duration ST in patients with severe impairment. Long-duration OT had a significant effect on FIM memory scores in the overall analysis, in the group with younger patients (55–64 years), and in the group of patients with the most severe dementia. Longer PT yielded no significant effects on the degree of improvement in FIM memory scores.

To confirm the validity of the standardized assessment of dementia level, we analyzed the data from 426 patients who also underwent MMSE. Spearman's correlation coefficient between the results of the two instruments was −0.591 (*p* < 0.001), confirming that dementia level was strongly associated with the MMSE score. The mean MMSE values were 25.7 (95% CI, 25.0–26.4) for patients with no deficit (normal scores in the evaluation of dementia), 21.4 (95% CI, 20.5–22.3) for patients with mild-to-moderate dementia, 14.8 (95% CI, 13.1–16.5) for patients with severe dementia, and 20.7 (95% CI, 17.4–24.0) for patients with unknown dementia level.

## 4. Discussion

The present study showed that the degree of improvement in language abilities was higher for patients who underwent long-duration ST than for those who underwent short-duration ST, suggesting that ST duration indeed affects the degree of improvement in language impairment as a sequela of stroke. Longer ST had an overall positive influence on language ability (FIM scores for comprehension and expression), with the greatest effect on comprehension. Additionally, OT and PT had a partial influence on language improvement, but the overall effect of OT and PT was less pronounced than that of ST.

Bhogal et al. [27] compared short- and long-duration ST groups in terms of the scores for the Porch Index Communicative Ability and the Token Test for assessing aphasia and found that the total training duration was significantly associated with the extent of improvement. The results of our study also indicate that a longer training time allowed for repetitive training and preparation of an environment that reinforces and enhances neural pathways [28].

Among younger patients, the influence of longer ST was greater than that noted in the older patients, who did not show significant differences between the effects of short- and long-duration treatment. Kelly-Hayes et al. [29] reported that the degree of disability and limitations in cognitive, physical, affective, and social domains after a stroke were greater in older patients. The results of the present study show that, among older patients, longer ST (or longer OT) were not associated with better outcomes. Thus, mostly the younger patients (<64 years) benefitted from the prolonged training time.

In the present study, we used the standardized assessment manual for elderly [21] to assess dementia level. This scale has proven adequate for assessing dementia, which was confirmed in the present study based on the significant correlation of dementia levels with MMSE scores. Among the patients with more severe dementia, the effects of longer ST duration were consistent; that is, patients with more severe dementia benefitted from the longer ST in terms FIM scores for both comprehension and memory. Belleville et al. [30] confirmed that memory training in patients with mild dementia produced changes in brain activation, as confirmed using functional magnetic resonance imaging, suggesting that these patients can retain some brain plasticity. Patients with more severe dementia who had a stroke, such as the patients in the present study, might be able to recruit brain regions outside the area affected by stroke [31, 32]. The results of the present study therefore emphasize that active training after stroke is worthwhile even in patients with dementia complications.

Overall, we found that, compared to OT or PT, ST improved aphasia to a greater extent. In this study, we used OT and PT as control variables. OT is usually prescribed to improve activities of daily living [33, 34], while PT is prescribed to improve movement [35]. Both interventions stimulate cognitive and language abilities because they require basic communication between patient and therapist. OT and PT also improve attention and concentration [33, 36]. Thus, OT and PT can contribute to communication training, and, by combining ST with OT and PT, we expect that patients may achieve more pronounced improvements in language and communication after stroke.

We employed inverse probability weighting after creating propensity scores using logistic regression analysis. Another option for analysis is the use of propensity score matching. However, we did not employ this method because it is nearly impossible to match between identical trend scores. If there is an imbalance in the sample of each group, then large amounts of data from one of the groups might be lost. Estimation with regression analysis requires trend scores and objective variables to be in a linear relationship; however, there is no reason to assume that propensity scores themselves would have a linear distribution. Inverse probability weighting is a technique that was developed to overcome these issues. This technique offers the ability to determine each group's marginal standard error and to have higher precision adjustments than those achievable in propensity score matching. While the analyses were performed on the initial assumption that only patients likely to improve with long-duration rehabilitation training were included, the use of propensity scores followed by the use of inverse probability weighting clarifies such assumptions.

The present investigation was a large-scale study using data regarding more than 3,500 patients treated in various hospitals and settings. Of the three previous studies that reported a beneficial effect of ST [37–39], one included 100 patients, one 30 patients, and one only 24 patients. Comparatively, the present data were collected from a large database and from medical records and therefore were considered to be relatively accurate and more representative of the characteristics of poststroke patients with aphasia. As Brady et al. concluded in their recent Cochrane Review [4] and as Cherney [8] emphasized, conclusive findings on the effects of dose and duration of ST are sparse and inconsistent. Moreover, to our knowledge, the present study is the first to investigate the independent effect of ST, OT, and PT duration on the degree of improvement in language impairment in Asian patients and the first study to apply such an analysis in subgroups stratified by age and dementia level. Moreover, our study involving a large sample of an Asian population is valuable because the prevalence of stroke has been reported to be higher in China and Japan [40] than in North America or Europe.

The present study had several limitations. First, we did not have access to data regarding certain relevant confounding factors including lesion location as well as prestroke educational history, occupational history, and language level. Therefore, it was impossible to adjust for these confounding factors. Second, language abilities were assessed using three items from the FIM instrument, and no formal, extensive language assessment was conducted. Finally, the specific modalities and delivery of the various ST, OT, and PT interventions were not controlled for. Further studies are needed to overcome these limitations.

## 5. Conclusions

Compared to short-duration ST, long-duration ST may provide better improvement in language impairment as a sequela of stroke. OT and PT may provide additional benefit regarding the improvement in communication abilities, but their effectiveness is less pronounced than that of ST. With respect to specific groups of patients, younger patients (<64 years) and those with more severe dementia benefit the most from undergoing long-duration ST instead of short-duration ST. Therefore, when evaluating the therapeutic strategy for aphasia patients after stroke, longer duration of ST should be considered especially in these groups of patients.

## Figures and Tables

**Table 1:** Demographic and clinical characteristics of the patients.

Characteristic	Category	Total	%
		(*N* = 3,551)	
Age	≥75 years	1,373	38.7
	65–74 years	956	26.9
	55–64 years	705	19.9
	≤54 years	517	14.6
Sex	Male	2,054	57.8
	Female	1,497	42.2
Dementia level	Normal	875	24.6
	Mild-to-moderate	1,063	29.9
	Severe	878	24.7
	Unknown	735	20.7
Stroke classification	Cerebral infarction	1,826	51.4
	Intracranial hemorrhage	824	23.2
	Subarachnoid hemorrhage	188	5.3
	Other	47	1.3
Duration of hospitalization	≤91 days	1,764	49.7
	≥92 days	1,787	50.3
Paralysis side	Right	1,207	34.0
	Left	1,092	30.7
	Unknown	1,253	35.3

Some data were missing; therefore, summing the number of patients in each category based on a specific characteristic might not equal the total number of patients in the study sample.

**Table 2:** Influence of training duration on the degree of improvement in Functional Independence Measures (FIM) score for comprehension.

Covariate	ST			OT			PT		
	OR	95% CI	*p*	OR	95% CI	*p*	OR	95% CI	*p*
Overall	1.361	1.098–1.687	0.005	1.590	1.036–2.441	0.034	1.204	0.782–1.852	0.399
Age									
≥75 years	1.016	0.710–1.454	0.931	1.484	0.721–3.055	0.284	1.251	0.675–2.318	0.477
65–74 years	1.251	0.850–1.842	0.257	1.225	0.643–2.332	0.538	1.611	0.766–3.388	0.209
55–64 years	2.356	1.382–4.016	0.002	3.205	1.535–6.690	0.002	1.865	0.923–3.767	0.082
≤54 years	1.758	1.100–2.812	0.018	1.295	0.362–4.634	0.691	0.430	0.137–1.350	0.148
Dementia level									
Normal	1.083	0.692–1.694	0.728	1.171	0.496–2.761	0.719	1.258	0.677–2.338	0.468
Mild-to-moderate	1.210	0.841–1.741	0.303	1.915	0.865–4.239	0.109	1.363	0.721–2.576	0.341
Severe	1.646	1.082–2.503	0.020	1.699	0.878–3.290	0.116	1.729	0.662–4.517	0.264

ST, speech-language-hearing therapy; OT, occupational therapy; PT, physical therapy; JCS, Japan Coma Scale; FIM, functional independence measure; OR, odds ratio; CI, confidence interval.

Adjustment for age, sex, hospital, hospitalization year, dementia level at hospitalization, JCS score at hospitalization, FIM walk/wheelchair score at hospitalization, FIM comprehension at hospitalization, number of days spent in hospital, and side with more severe paralysis. Further adjustment for OT and PT in the analysis of ST, ST and PT in the analysis of OT, and OT and ST in the analysis of PT. Each OR represents the comparison between long and short therapy duration, with the OR for short duration always being reference.

**Table 3:** Influence of training duration on the degree of improvement in Functional Independence Measures (FIM) score for expression.

Covariate	ST			OT			PT		
	OR	95% CI	*p*	OR	95% CI	*p*	OR	95% CI	*p*
Overall	1.251	0.999–1.567	0.051	1.399	0.918–2.131	0.119	1.404	0.849–2.322	0.186
Age									
≥75 years	1.041	0.704–1.539	0.842	1.220	0.600–2.480	0.583	2.102	1.245–3.548	0.005
65–74 years	1.108	0.745–1.649	0.612	0.896	0.483–1.662	0.728	1.192	0.473–3.005	0.709
55–64 years	1.177	1.031–3.062	0.038	1.470	0.591–3.658	0.407	2.154	0.970–4.784	0.060
≤54 years	1.664	1.045–2.650	0.032	4.686	1.925–11.405	0.001	0.363	0.111–1.192	0.095
Dementia level									
Normal	1.102	0.683–1.778	0.691	1.133	0.514–2.499	0.756	1.850	0.895–3.821	0.097
Mild-to-moderate	1.130	0.786–1.624	0.509	1.720	0.794–3.726	0.169	2.090	1.139–3.834	0.017
Severe	1.314	0.843–2.047	0.228	1.495	0.775–2.883	0.230	1.622	0.599–4.393	0.341

ST, speech-language-hearing therapy; OT, occupational therapy; PT, physical therapy; JCS, Japan Coma Scale; FIM, functional independence measure; OR, odds ratio; CI, confidence interval.

Adjustment for age, sex, hospital, hospitalization year, dementia level at hospitalization, JCS score at hospitalization, FIM walk/wheelchair score at hospitalization, FIM expression at hospitalization, number of days spent in hospital, and side with more severe paralysis. Further adjustment for OT and PT in the analysis of ST, ST and PT in the analysis of OT, and OT and ST in the analysis of PT. Each OR represents the comparison between long and short therapy duration, with the OR for short duration always being reference.

**Table 4:** Influence of training duration on the degree of improvement in Functional Independence Measures (FIM) score for memory.

Covariate	ST			OT			PT		
	OR	95% CI	*p*	OR	95% CI	*p*	OR	95% CI	*p*
Overall	1.087	0.876–1.350	0.449	1.567	1.029–2.385	0.036	1.137	0.708–1.825	0.596
Age									
≥75 years	0.879	0.620–1.246	0.468	1.324	0.672–2.606	0.417	1.404	0.779–2.530	0.259
65–74 years	0.936	0.616–1.423	0.758	1.119	0.607–2.063	0.718	1.044	0.464–2.348	0.917
55–64 years	1.533	0.950–2.476	0.080	3.368	1.586–7.152	0.002	1.368	0.594–3.151	0.461
≤54 years	1.690	1.001–2.853	0.050	1.979	0.660–5.932	0.223	0.576	0.176–1.884	0.361
Dementia level									
Normal	0.834	0.538–1.292	0.416	0.831	0.383–1.804	0.639	1.544	0.749–3.221	0.236
Mild-to-moderate	0.872	0.591–1.287	0.489	1.528	0.699–3.338	0.288	1.444	0.787–2.649	0.236
Severe	1.653	1.092–2.502	0.017	2.170	1.233–3.821	0.007	1.166	0.445–3.057	0.755

ST, speech-language-hearing therapy; OT, occupational therapy; PT, physical therapy; JCS, Japan Coma Scale; FIM, functional independence measure; OR, odds ratio; CI, confidence interval.

Adjustment for age, sex, hospital, hospitalization year, dementia level at hospitalization, JCS score at hospitalization, FIM walk/wheelchair score at hospitalization, FIM memory at hospitalization, number of days spent in hospital, and side with more severe paralysis. Further adjustment for OT and PT in the analysis of ST, ST and PT in the analysis of OT, and OT and ST in the analysis of PT. Each OR represents the comparison between long and short therapy duration, with the OR for short duration always being reference.
